# A machine learning based empathy mapping framework for enhancing user experience through app review analysis

**DOI:** 10.1038/s41598-025-30729-4

**Published:** 2025-12-02

**Authors:** Faryal Ishfaq, Safdar Nawaz Khan Marwat, Waseem Ullah Khan, Sara Shahzad, Shahid Khan, Qammer H. Abbasi

**Affiliations:** 1 Department of Computer Science, University of Peshawar, Peshawar, Pakistan; 2 Department of Computer Systems Engineering, University of Engineering and Technology, Peshawar, 25120 Pakistan; 3 Secured IoT Devices Lab, University of Engineering and Technology, Peshawar, 25120 Pakistan; 4 COMSATS University Islamabad, Abbottabad Campus, Abbottabad, Pakistan; 5 James Watt School of Engineering, Glasgow, G12 8QQ UK; 6 Distinguished Visiting Research Fellow, Abu Dhabi University’s College of Engineering, Abu Dhabi, UAE

**Keywords:** Software engineering, User experience, Empathy mapping, Deep learning, Natural language processing (NLP), Text classification, Sentiment analysis, BERT, App review mining, Topic modeling, Engineering, Mathematics and computing

## Abstract

The effectiveness of software applications largely depends on the user experience (UX), since it has a direct impact on user engagement and satisfaction. Empathy mapping is an important design thinking technique that organizes user perceptions into distinct categories for better understanding. However, traditional empathy mapping methods rely entirely on interviews and manual analysis which are both time-consuming and costly, thereby limiting the scalability of UX design and research. To address these challenges, this study presents an automated process for empathy mapping by analyzing user-posted app reviews. This study uses the Bidirectional Encoder Representations from Transformers (BERT) model for sentiment analysis, classifying user reviews as either positive (gain points or desires) or negative (pain points or frustrations). Latent Dirichlet Allocation (LDA) is then used to apply topic modeling to pinpoint preferences and important themes. By concentrating on gains and pains, this method automates the traditional manual and costly process of design thinking and empathy mapping, making it more scalable and efficient through data-driven insights. In training, the proposed model with several versions of BERT model, the binary accuracy improved from 78.14 to 98.61%, with precision achieving 97.82%, F1 score of 98.62%, and recall up to 99.42%. The validation accuracy also increased from 87.40 to 92.58%, with an F1 score 92.59%, precision of 92.43%, and recall of 92.75%. These accurate results indicate that the proposed model may be used by user experience design teams, which will help them improve and streamline UX design while also assisting developers in promptly receiving user feedback.

## Introduction

In recent years, software engineering researchers have shown increasing interest in User Experience (UX), particularly in addressing emotional needs like enjoyment, motivations such as helping others, and values like environmental care. Understanding how these elements influence UX design is essential for creating more effective user experiences and contributing to the overall success of software. A well-designed user experience offers clear navigation paths within a software application, provides easy access to information, and enables users to achieve their goals with minimal effort^1^.

This research addresses the resource limitations faced by low-budget software projects while carrying out requirements analysis and evaluating user experience. The aim is to develop a system that identifies key tasks for software applications, providing a practical alternative for projects that cannot afford to hire professional services for problem analysis and user interviews. By focusing on empathy and analyzing app reviews, the goal is to improve software development and functionality by aligning better with user sentiments & needs. This research proposes cost-effective techniques to understand the opinions of users that will help in improving UX through user-centric design methods and well-defined functionalities. To further support this goal, this research introduces a comprehensive approach to analyzing user application reviews by combining topic modeling and sentiment analysis to create empathy maps. This approach captures the recurring themes (i.e., pain points and desired features) and emotional tone (positive or negative) in user feedback, providing software engineers with deeper and more valuable insights. These insights can guide feature prioritization, enable data-driven UX improvements, and contribute to the development of more engaging and successful software products. This methodology offers a holistic perspective on UX and directly aids companies in making informed decisions during app development. This research paves the way for practical advancements in engineering requirements practices, particularly for modern app development teams.

Human-centered design facilitates a deeper understanding of people’s needs and builds empathy for problems that a product or service intends to address^2^. Understanding the user requirements truly allows designers to shape products that meet user expectations. Using a clear approach in design thinking helps designers to pinpoint and convey user demands, resulting in long-term solutions that meet those needs^3^. Enhanced UX is important to the success of any software product. Empathizing is the initial phase of UX design and focuses on understanding real users’ needs to ensure a more effective and engaging interaction with the product^4^. Therefore, the overall success of software products deeply relies on the quality of the user experience^5^. Deeply understanding users’ needs enables designers to develop solutions that are better tailored to those needs and the broader system context. Design thinking frameworks provide tools for accurately identifying user requirements and communicating effectively. This results in solutions that align well with user expectations and improve interaction effectiveness. The five phases of the design thinking process typically include empathy, definition, ideation, prototyping, and testing. Notably, the empathy phase involves thorough research into users’ needs^6^.

In this context, focusing on UX is not just beneficial; it is essential. The success of software increasingly depends on how users perceive and experience the product. Products that are designed with users in mind are more likely to meet expectations and enhance users’ quality of life. Personas are essential tools in interaction design. They help identify and address problems with system flow during prototype testing and support user-centered development across different phases. In the requirements engineering phase, personas assist in defining user needs and provide insights into specific user requirements. During design, they help assess whether those needs are being met. In marketing, they guide targeted strategies based on user segmentation. Personas, derived from real user profiles, help maintain focus on specific user types throughout the design process. Social media platforms offer access to publicly available user information that reveals interests and lifestyles, enabling the development of rich personas. These personas assist in system testing to ensure functionality meets user requirements and also help customize marketing and content strategies according to user preferences^7^. Additionally, personas help development teams understand user group characteristics, guide feature design for primary users, and foster a stronger connection between the design team and the end user. However, persona techniques have limitations. They often rely on informal data, are difficult to implement practically, and may unrealistically represent users. Furthermore, personas are not a substitute for direct user research, which is critical for truly user-centered design^8^. Applying design thinking principles along with empathy mapping reveals users’ feelings, emotions, and motivations, enabling the development of solutions that meet their expectations and improve the overall experience.

**Fig. 1:**
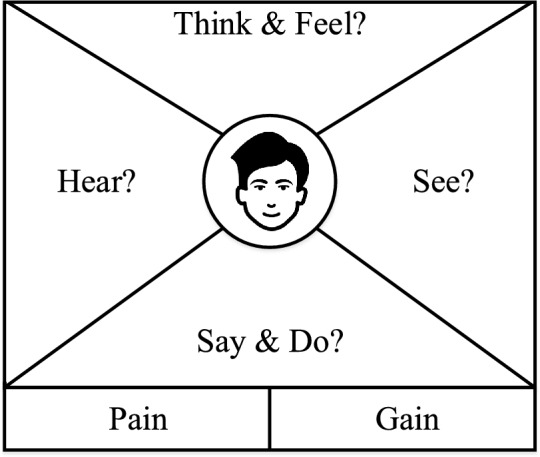
Empathy map template, representing six user dimensions (Think & Feel, Hear, See, Say & Do, Pain, and Gain).

To effectively solve user problems, designers must prioritize and actively consider user feedback over personal assumptions. Empathy in design requires a comprehensive understanding of users, not only their needs but also their challenges and the broader context in which they operate. Empathy should be maintained throughout every stage of the design thinking process. UX designers use this framework to understand users’ needs, emotions, and motivations. This understanding enables productive and meaningful interaction between the user and the system^9^. Empathy maps, as opposed to fictional personas, are based on the information gathered from customer segments or user groups. Gaining a clear understanding of user perspectives and pain points is essential for designing products that align with evolving expectations^10^. One design thinking method is the Empathy Map Method (EMM), a creative technique that aims to build empathy for users and gain new insights into their needs^2^. The first version of the empathy map, introduced by Scott Matthews, consisted of four distinct sections designed to build a thorough understanding of the user (refer to Fig. 1). While the traditional empathy map consists of four quadrants, i.e., says, thinks, does, and feels. Additional sections, such as Pains and Gains are often included to better understand the user’s frustrations and motivations. These insights can support the development of user personas by highlighting user reactions to specific updates, including which features are well-received and which pose usability challenges. Furthermore, pain points can be analyzed at multiple UX levels, such as the interaction level, journey level, and relationship level, allowing UX designers to better target areas for improvement. Subsequently, Bland^11^ expanded the empathy map framework by adding new segments, specifically the Pain and Gain areas. The updated model now consists of six sections: Think and Feel—the thoughts and emotions experienced by the user; See—what the user notices in their surroundings; hear—the environmental influences on the user; Say & Do—what the user communicates and how they behave in public; Pain—risks and difficulties the user faces; and Gain—outcomes the user seeks and actions required to achieve them^4^.

The emergence of Electronic Word of Mouth (eWOM) is closely tied to the rise and widespread adoption of the internet. It is regarded as one of the most influential forms of informal media for businesses, playing a significant role in customers’ decision-making processes and holding a strong position among enterprises as a channel of influence^12^. With the continued expansion of e-commerce, customer reviews and comments have gained increasing importance, directly shaping purchasing decisions^13^. Extensive research has been conducted on mobile app reviews collected from platforms such as the Google Play Store and Apple’s App Store. By examining feedback from these sources, developers can identify potential areas for product enhancement. Sentiment analysis of user comments is typically performed using Natural Language Processing (NLP) techniques and various Machine Learning (ML) algorithms. These analyses often classify reviews into categories such as “positive,” “negative,” or “neutral,” or assign a rating scale, for example, from 1 to 5 or − 1 to + 1. While such categorization is useful, it does not fully capture the emotions conveyed in a review. Beyond assigning ratings, emotion detection can provide more nuanced insights, identifying not only positive and negative sentiments but also specific emotions such as frustration, happiness, anger, and fear^13^. Text-Based Emotion Detection (TBED) has become a growing area in NLP focused on identifying emotions in written language using models from psychology^14^. Emotions provide insight into how customers feel about their experience with an app and can be analyzed to better understand their overall perception and satisfaction. During the design process, considering users or customers is essential, often through tools such as personas. These help designers understand user characteristics, artifacts, and needs, enabling them to develop solutions that effectively address those needs. The empathy map also supports the creation of personas by incorporating the contextual questions that assist and guide designers^15^. Every designer should incorporate empathy training into their approach. Designers must consider the emotional response of users when interacting with a product, not just its appearance. This focus is particularly crucial in a diverse user environment, where individuals may have different backgrounds, ethnicities, lifestyles, and even special needs. UX designers must therefore strive to understand and accommodate diverse user requirements. Emphasizing empathy strengthens relationships with users and improves elements such as tone, sentiment, and interaction management.

Existing empathy-mapping studies mainly depend on interviews or small datasets, which limit scalability and real-time insight generation. Few have explored automated empathy mapping using large-scale app reviews. This paper presents a practical and cost-effective approach that combines sentiment analysis and topic modeling for analyzing user reviews. The results are used to generate empathy maps that help software teams identify both functional requirements and emotional needs. By utilizing publicly available data, the proposed method offers a scalable and automated alternative to traditional user interviews, supporting the development of user-centric applications. This research integrates deep-learning-based sentiment analysis i.e., Bidirectional Encoder Representations from Transformers (BERT) and topic modeling such as Latent Dirichlet Allocation (LDA) to identify user pain and gain points automatically, providing a scalable and reproducible alternative to manual UX research. The remainder of this paper describes the methodology used, the experimental setup, model implementation, results, and how these findings contribute to creating gain and pain point summaries for design and development teams.

## Literature review

This section presents a comprehensive review of the literature relevant to this research. It explores key concepts such as UX, text analysis, app reviews, empathy mapping, and the application of machine learning, particularly BERT, in sentiment and emotion analysis. The discussion identifies research gaps that this study aims to address.

### User experience

UX plays a vital role in application and website design, going beyond mere functionality to include how users feel during interactions. With the rising importance of UX, many researchers and industries are focusing on it. UX is a scarce and expensive expertise, and many developers lack proper training in it. This leads to poor implementation of UX in software products^16^. Software engineering often simplifies UX, focusing only on task efficiency and neglecting users’ emotional responses^17^. UX involves emotions, perceptions, and attitudes that arise during interaction with digital products. Human–Computer Interaction (HCI) uses terms like “emotional design,” while software engineering emphasizes function and efficiency, leading to differing interpretations. HCI experts argue that these emotional aspects should be integral to product design. Incorporating UX practices helps in delivering useful and satisfying experiences. Tools like feedback analysis and empathy maps help understand users’ expectations. However, research shows a gap in integrating these UX tools in software development processes.

### Empathy mapping

An empathy map acts as a useful resource, offering detailed insights into users’ expressions, emotions, actions, and thoughts while engaging with the app^6^, and supporting the development of business models from the clients’ perspective. Empathy is a cornerstone of the design thinking process because it facilitates emotional connections with users. Tools such as empathy maps and personas offer vital insights into users’ feelings, motivations, and beliefs^14^. Through the EMM, designers can gain a better understanding of user perspectives. This knowledge contributes to creating engaging interactions and business strategies that are well-aligned with user needs and expectations^2^.

Even though the empathy map template looks easy to use, it requires specialized skills to collect the data for each of its six sections & analyze it to achieve the necessary level of user awareness. Since UX expertise is limited, it often comes at a higher cost. Recent developments in UX design and design thinking have led experts to explore and utilize Artificial Intelligence (AI) techniques to automate the manual process of creating and analyzing empathy maps. Several studies have been conducted that utilize AI techniques to automatically extract, interpret, and process information from unstructured documents, including methods such as requirement mining to identify relevant user needs^18^. A study published in March 2024^19^ explored the automation of empathy maps using machine learning by organizing interview data into the four traditional quadrants: “Feels”, “Says”, “Does”, and “Thinks”. However, there has been little to no research aimed at identifying users’ pain points and gain points or at automating empathy map creation using app reviews to improve UX. The present research concentrates on the pain and gain quadrants to capture what users think and feel about the app.

### Text analysis

Text analysis enables the extraction of requirements from unstructured textual feedback. Topic modeling techniques like LDA have been used to identify non-functional requirements and latent themes in app reviews^20,21^. Studies such as^22,23^ apply aspect-based sentiment analysis to detect and categorize fine-grained user opinions about specific features, enabling more actionable insights for product development^24,25^. Automatic classification models within review analysis frameworks help filter irrelevant reviews and improve the signal-to-noise ratio in user feedback mining, supporting deeper insight generation through topic and sentiment analysis^26^.

### App reviews

Unsupervised sentiment classification techniques have made it possible to extract meaningful emotional insights without heavy training data, enabling scalable analysis of user opinions^27^. App reviews are a rich, unsolicited source of user feedback at scale. They capture sentiments, feature requests, and usability concerns. Automated techniques help reduce manual effort by prioritizing feedback on bugs, enhancements, and new features^28^. However, their unstructured nature and informal language present analytical challenges. Automated classification and clustering methods have been proposed to categorize reviews into actionable groups such as bug reports, enhancement suggestions, and UX feedback^29^. Accurate classification of user-reported issues, similar to automated bug classification in software engineering, can significantly enhance feedback triage and usability analysis when applied to large-scale app reviews using deep learning-based NLP techniques. Recent studies also show that customizing models like BERT for domain-specific sentiment analysis, such as Stack Overflow posts, can yield significantly improved performance over traditional tools, making them more practical for software engineering applications^30,31^.

User reviews and comments can provide valuable insights into user experiences with app updates, helping identify the user’s pains and gains. Sentiment analysis and emotion detection techniques further support this process by gauging user satisfaction across different updates and versions^32,33^. Designers can utilize machine learning to identify patterns in complex information, supporting tasks such as interpreting semantics from speech and text to enhance user experience across various types of interactions^34^. Machine learning can learn from data to recognize patterns, predict outcomes, and make decisions, making it useful for understanding natural speech, adapting interfaces, and enhancing real-time user interactions^35–38^. In natural language processing, deep learning, especially BERT, has improved tasks such as emotion detection and sentiment analysis.

### BERT

BERT uses bidirectional attention and a pre-trained transformer architecture for better contextual understanding. It is trained on massive corpora and fine-tuned for specific tasks, outperforming models like Recurrent Neural Networks (RNNs), Long Short-Term Memory (LSTM), and Bidirectional Long Short-Term Memory (BiLSTM) in classification, question answering, and completion tasks. BERT, built on transformer architecture, uses feed-forward layers and multi-head attention instead of recurrent units such as LSTM and Gated Recurrent Unit (GRU). Through self-attention, it connects words across different positions to generate contextualized representations, enabling more accurate interpretation of the input text^31^. Recent studies using BERT-based models for sentiment analysis on developer forums like GitHub, Jira, and Stack Overflow have shown clear improvements over earlier tools, with notable gains in accuracy ranging from 6 to 12%^39^.

Requirements Engineering using Bidirectional Encoder Representations from Transformers (RE-BERT) outperforms traditional models like Support Vector Machine (SVM), Convolutional Neural Networks (CNN), LSTM, and GRU in extracting user requirements from noisy app reviews^23^. This opinion mining approach uses fine-tuned BERT to extract accurate and context-sensitive software requirements from unstructured text^40^. BERT also outperforms BiLSTM in both accuracy and recall^41^. Ensemble models like EmoDet2 (BERT + BiLSTM) have shown high F1-scores in emotion detection tasks (0.75 on SEMEVAL-2019)^32,42^. In Ref.^43^, the achieved F1-score was 89% on tweets. BERT-based classifiers outperform LSTM in classifying app reviews and identifying issues. Unlike recurrent models, BERT uses self-attention to capture long-range context^44^. Applications include bot detection, sentiment classification on social media, and domain-specific language models like BERT-Base (110M parameters) and BERT-Large (340M)^43^. BERT also improves performance in software engineering tasks, such as bug prediction and API recommendation, compared to traditional tools like SentiStrength-SE^39^.

### Sentiment analysis

Sentiment analysis is used in software engineering to classify user feedback from reviews and forums as positive, negative, or neutral. Effective sentiment analysis relies on factors like data quality, word selection, and classification methods^45^. In recommendation systems, identifying sentiment adds complexity beyond recognizing general content, as it requires understanding a user’s opinion about a specific item, such as whether a recommended movie is truly worth watching^46^. Lexicon-based methods often struggle with sarcasm, negation, and implicit sentiment, which modern models like BERT can handle more effectively. Transfer learning, where models are pre-trained and fine-tuned for specific tasks, has become a key technique in NLP, enabling text-to-text frameworks and improving performance across applications^47,48^.

Semantic analysis techniques, including LDA and deep learning models, are increasingly used to mine and classify customer requirements for the conceptual design of informed and innovative products^8^. Recent studies demonstrate that BERT performs effectively across a range of applications, improving predicted accuracy in areas like social media bot detection and feature extraction. In sentiment classification, BERT enables researchers to label tweets as negative or positive without relying on topic modeling techniques such as LDA, ensuring that extracted features are not limited to topic-specific contexts. Because of its bidirectional attention mechanism, which provides richer contextual understanding, it makes it particularly suitable for sentiment analysis. These capabilities also allow BERT to be integrated with artificial neural network models, further illustrating its adaptability and strength in addressing text-based challenges^37^. Fine-tuning pre-trained models like BERT has shown better sentiment classification results in software engineering tasks compared to traditional domain-specific tools^49^.

With the rise of social media, sentiment and emotion analysis have become vital for interpreting unstructured text data and understanding human expression at scale^50^. Emotion detection builds on sentiment analysis by identifying specific feelings such as anger, joy, or surprise. Natural Language Processing methods, despite lacking facial or vocal cues, can effectively detect emotional signals. Sentiment analysis tools not tailored for software engineering perform poorly due to domain gaps. Hybrid models combining deep learning and machine learning have proven effective for emotion recognition in text, addressing limitations of earlier keyword and lexicon-based approaches^51^. Fine-tuned models like BERT achieve higher accuracy by learning contextual cues. The Text Filtering Method (TFM) has shown improvements in detection accuracy across classifiers. Some models also align review sentiment with star ratings and visualize key insights to aid decision-making^44^. Emotion detection identifies discrete emotional states from user-generated content. ML-based classifiers have extracted emotions from hotel reviews to improve service quality. Some studies benchmark performance on emotions such as anger, joy, or trust using machine learning^52^. Others detect emotion presence or absence rather than specific categories. Hybrid methods combining ML and Deep Learning (DL) translate review data into emotion vectors to enhance emotion classification in datasets such as tweets and dialogues. Mapping user emotions to service feedback helps organizations better meet customer expectations and improve satisfaction. The current study also addressed deep learning in both the physical and engineering realms, going beyond UX and sentiment analysis studies. To increase heat transfer efficiency, the authors in Refs.^53,54^ used neural models with the hybrid nanofluids, whereas authors in Refs.^55,56^ employed the Artificial Neural Network (ANN) and computational fluid dynamics (CFD) techniques for thermal and bioconvective study.

### Comparative sentiment analysis of app reviews

Recent studies emphasize the effectiveness of deep learning and transformer-based models in carrying out sentiment analysis on app reviews. In Ref.^57^, LSTM outperformed Artificial Neural Network (ANN) and SVM on 33,000 Google Play Store reviews, achieving 92.75% validation accuracy and a 96.38% F1-score. Similarly, BERT surpassed traditional models like Logistic Regression, SVM, Naïve Bayes, Ridge Classifier, and Voting Classifier in Ref.^58^, with 93.87% accuracy on the IMDB dataset. Transformer-based models were further evaluated in Ref.^59^, where BERT, DistilBERT, RoBERTa, and XLM-RoBERTa were applied to Spotify reviews. DistilBERT achieved the highest accuracy (71.68%), while XLM-RoBERTa had the best F1-score (69.24%). In Ref.^60^, RoBERTa-base and RoBERTa-large outperformed others on the Naver dataset for ABSA, with 97.62% accuracy and a 94.77% F1-score. DistilBERT also performed well (96.83% accuracy, 91.96% F1). In contrast, GPT-4 and GPT-3.5-turbo lagged behind (86.95% accuracy, 63.94% F1), and traditional models like Bi-LSTM, LSTM, and RNN scored lower F1-scores of 63.16%, 47.45%, and 61.04%, respectively. As shown in Table 1, transformer models like BERT consistently outperform traditional methods, offering superior contextual understanding and more reliable sentiment extraction in app review analysis.

**Table 1:** Comparison of sentiment analysis models on user reviews.

Paper/study	Dataset used	Model(s) applied	Accuracy	F1-Score	Notes
Samanmali et al.^57^	Google Play App Reviews of 15 popular Apps	Logistic Regression, Naïve Bayes, SVM	$\sim$ 92.75%	96.3%	LSTM outperformed ANN and SVM with the highest accuracy
González et al.^58^	IMDB movie reviews	BERT, Logistic Regression, SVM, Naïve Bayes	93.87%	Not provided	BERT outperformed all models
Eser & Sahin^59^	Spotify App Reviews (Google Play Store)	BERT, DistilBERT, RoBERTa, XLM-RoBERTa	71.68 (DistilBERT)	69.24 (XLM-RoBERTa)	DistilBERT achieved highest accuracy; XLM-RoBERTa had best F1 score
Perikos & Diamantopoulos^60^	Naver Dataset	RoBERTa, DistilBERT, XLNet	RoBERTa-large (97.62%)	RoBERTa-large (94.77%)	RoBERTa-large outperformed other models, showing superior accuracy and F1 score

### Topic modeling

As software and mobile apps evolve, some update frequently while others lag. Users often compare updates across multiple apps and may become dissatisfied when one falls behind. User reviews highlight such issues and reveal problems developers might overlook. Research has developed automated frameworks to analyze app reviews using topic modeling to identify key themes within user comments^26^. These systems perform aspect-based sentiment analysis on user reviews, extracting semantic topics and sentiment polarity for each aspect to provide structured insights into areas needing improvement in mobile apps^61^. Joint topic-sentiment analysis explores the connection between topics and the sentiments expressed, identifying which aspects are associated with positive or negative opinions. Broader attitudes, such as political orientations or platform preferences, are captured through viewpoint and perspective identification^40^.

Topic modeling extracts recurring themes or subjects from reviews, offering insights into user experiences, preferences, and pain points. This guide’s development prioritizes enhancements and improves satisfaction. It categorizes unstructured reviews into themes, helping developers understand concerns, address issues, and discover unstated frustrations. Its main phases include data representation, latent topic decomposition, and topic extraction. Latent topic decomposition uncovers themes using matrix factorization or probabilistic models like LDA. Topic extraction links topics to specific words and texts. LDA is widely used for analyzing user comments. Studies emphasize its role in uncovering hidden themes and extracting features from feedback for further analysis and improvement^52^.

### Text summarization

Text summarization condenses long content while retaining the main points, useful for identifying key ideas efficiently^62–64^. It is applied in news, research, social media, and customer service^64^. There are two categories: abstractive, which generates new phrases with the same meaning, and extractive, which chooses significant sentences from the source text. Tools like Pegasus and T5 perform well on benchmark datasets^65,66^. ChatGPT-4 has shown strong performance in NLP tasks such as text summarization, data analysis, and question answering, delivering higher accuracy and coherence compared to other Large Language Models (LLMs)^67,68^. Word-to-sentence generation constructs coherent sentences from unordered lists of words or topic phrases, often extracted via topic modeling methods^64,69^. It enhances interpretability for non-technical stakeholders. It organizes unordered words into grammatically correct, meaningful sentences using language structure rules^70^. This is used when turning random words into readable text. The methods in this study emphasize the importance of understanding user emotions and sentiments to enhance UX. Reviews, when processed via sentiment analysis and empathy mapping, offer insights into user needs. Leveraging these approaches helps improve app quality, usability, and user-centered design. Earlier studies typically examined sentiment analysis or empathy mapping in isolation. In contrast, this research integrates the two by applying BERT and LDA to social media app reviews, enabling automated empathy map generation from large-scale user feedback, an approach that, to the best of our knowledge, has not been systematically explored before.

## Materials and methods

**Fig. 2:**
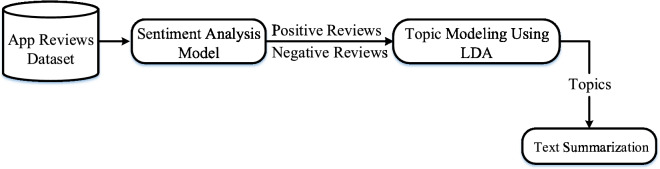
Stepwise flow of research methodology showing app reviews data processing, sentiment analysis model, topic modeling using LDA and text summarization.

**Fig. 3:**
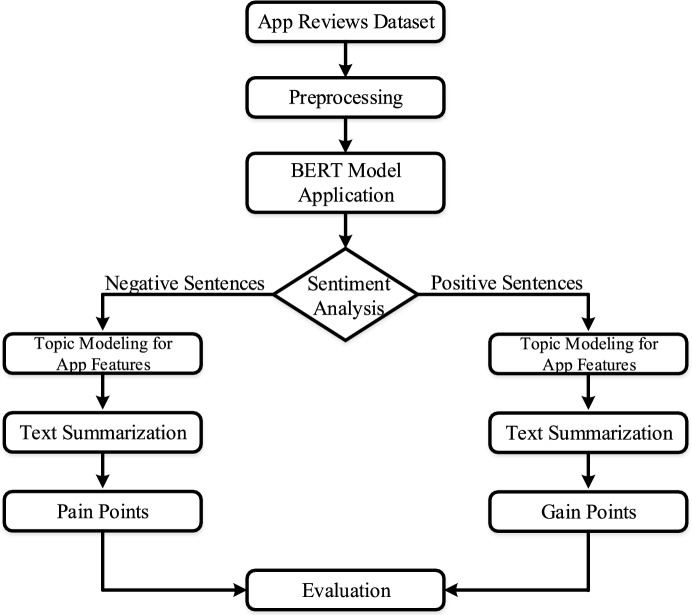
Process workflow of the proposed model illustrating key phases from data preprocessing to analysis and performance evaluation.

This study aims to analyze user-generated reviews from Instagram and Threads to extract meaningful feedback through sentiment analysis and topic modeling. These methods help identify pain points and gain points to support UX improvements. Figure 2 illustrates the overall research methodology, while Fig.  3 presents the detailed workflow.

### Data acquisition

User reviews in the form of comments were extracted from the Google Play Store to identify problems users face, their frustrations (pain points), and appreciated features (gain points). These reviews are crucial for understanding real-time user experience and for improving app usability and satisfaction. The user-generated data serves as a valuable source of both qualitative and quantitative feedback, reflecting the emotional and functional aspects of UX design. The datasets of Instagram app reviews^71^ from the App Store and Google Play Store, as well as the Threads app reviews dataset^72^ by Saloni Jhalani, were used. Both datasets were obtained from https://www.kaggle.com and contain anonymized user reviews originally collected from the respective app stores. The data were used solely for research purposes under Kaggle’s open data license, in accordance with the terms of service of both platforms and institutional ethical guidelines for secondary data analysis. Three progressively larger datasets were created, as shown in Table  2.

**Table 2:** Overview of datasets used for sentiment analysis experiments.

Dataset	Total reviews	App source(s)	Composition	Purpose
Dataset 1	10,000	Instagram	All reviews from Instagram	Initial fine-tuning of smaller BERT
Dataset 2	20,000	Instagram & Threads	10,000 from Instagram, 10,000 from Threads	Main experiments (Standard BERT-Base & RoBERTa)
Dataset 3	40,000	Instagram & Threads	20,000 from Instagram, 20,000 from Threads	Evaluation of large-scale performance

### Data labeling

To prepare for binary sentiment classification, reviews with 1–2 stars were labeled as Negative (0), while those with 4–5 stars were labeled as Positive (1). Reviews rated with 3 stars were excluded to avoid ambiguity and simplify the task, following conventions in existing literature^73,74^ as mentioned in Table  3. The large dataset size (over 200,000 reviews) allowed this exclusion without reducing data diversity. An 80:20 ratio was used for splitting training and validation sets.

**Table 3:** Categorization of app review ratings into positive and negative.

Rating	Label	Category
1, 2	0 (Negative)	Negative
3	Excluded	Not categorized
4, 5	1 (Positive)	Positive

### Data preprocessing

A comprehensive preprocessing pipeline was implemented to ensure data quality and compatibility with transformer-based models. All text was lower-cased to maintain consistency with uncased BERT variants. Punctuation, emojis, and non-word characters (e.g., @, #, %) were removed to minimize noise, along with digits irrelevant to sentiment interpretation. Stopwords (e.g., “and,” “the”) were removed to retain only meaningful content. Lemmatization was applied to reduce inflected forms to their dictionary base, ensuring consistency across similar terms. Stemming was also used to generalize words to their root forms. Reviews that were extremely short or excessively long were filtered out, and missing values were addressed to maintain dataset quality. Both stemming and lemmatization were tested to assess their impact on text normalization. Lemmatization was chosen for the final model because it preserved contextual meaning and improved classification accuracy, while stemming was retained only for comparison. These steps prepared the reviews for optimal learning during model training by simplifying input while preserving semantic content.

## Sentiment analysis methodology

Tokenization, which divides sentences into smaller parts like words or subword tokens, is an essential preprocessing step. This study employs subword tokenization, which effectively handles rare or complex words by dividing them into smaller units. This ensures essential context and meaning are preserved, supporting accurate sentiment and topic analysis. To understand textual and contextual information effectively, this research utilizes the deep learning-based BERT model, which has been widely adopted for emotion and sentiment detection in user-generated content such as tweets and app reviews^35,36^. Prior studies have shown that BERT consistently outperforms baseline models in emotion detection, achieving higher accuracy than other approaches^33,34,75^.

**Fig. 4:**
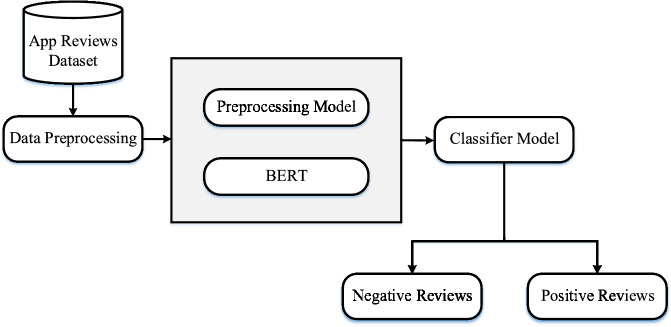
Stepwise workflow process of sentiment analysis illustrating data preprocessing, model training and validation, classification of sentiment polarity into positive and negative categories.

In this research, BERT is applied to classify app reviews into positive and negative sentiments. A classifier processes the labeled dataset to identify each review’s sentiment. Topic modeling is then applied separately to each sentiment class: positive reviews are analyzed to extract gain points, while negative reviews reveal pain points for empathy mapping. BERT, a transformer-based model, excels at tasks like text classification and sentiment analysis. To evaluate performance, different versions of BERT are tested. Among them, a smaller BERT model, consisting of 4 transformer layers, 8 attention heads, and a 512-dimensional hidden size, is employed for its computational efficiency. This uncased model treats uppercase and lowercase text equally, making it well-suited for low-resource environments while maintaining reliable performance in sentiment classification tasks. The overall sentiment analysis process is illustrated in Fig. 4. The sentiment analysis module used BERT to classify reviews as positive or negative, and the topic modeling module used LDA to identify key discussion themes, which combined provided both emotional and thematic insights into user feedback, forming the basis of the automated empathy mapping process.

### Topic modeling

**Fig. 5:**
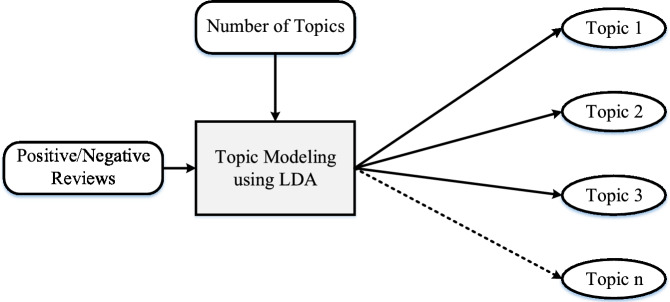
Key phases of topic modeling employing LDA used for the extraction and visualization of topics from app reviews data.

Topic modeling is applied to both positive and negative review classes. LDA is used to uncover hidden themes across the review corpus. LDA assumes that each document may contain multiple topics and that each topic is characterized by a distribution over words. The LDA model groups terms that frequently appear together into coherent themes. For example, themes may emphasize features such as “filters”, “DMs”, and “stories”, or problems like “lag”, “freeze”, and “crash”. The topic modeling process is visualized in Fig. 5. To improve topic coherence and clarity, preprocessing steps include the removal of irrelevant elements like stopwords and punctuation, thereby structuring the input data more effectively.

### Sentence summarization and generation

**Fig. 6:**
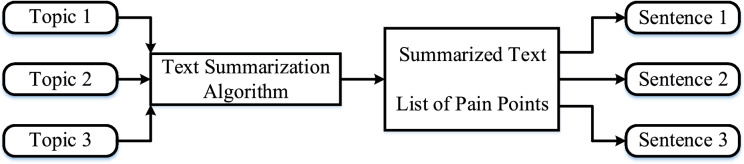
Conversion of topic outputs into concise, interpretable sentences using the text summarization algorithm.

Following sentiment analysis, the reviews were divided into positive and negative groups. Topic modeling was applied separately to each group to uncover the main discussion themes. Reviews associated with positive sentiments were labeled as “gain” points, reflecting satisfaction, appreciation, or useful features, while those linked to negative sentiments were labeled as “pain” points, indicating usability issues or frustrations. To convert topic keywords into understandable sentences, sentence summarization and generation techniques were employed. This approach transforms keyword-level topic outputs into meaningful, human-readable statements that are suitable for UX designers and decision-makers. The overall process is depicted in Fig. 6. Word-to-sentence generation involves arranging keywords into complete, grammatically correct sentences using natural language generation (NLG) models such as RNNs, Markov Models, and GRUs. In this research, Paperguide and Scribbr were selected for extractive summarization because they provide clear methods and allow references to be traced, ensuring that the summaries can be reproduced reliably. ChatGPT (GPT-3.5) was used for abstractive summarization, as it demonstrates strong contextual understanding and produces coherent, reader-friendly summaries.

Due to resource constraints, large-scale summarization models like T5 and Pegasus were not fine-tuned. Instead, API-based generation and manual curation were adopted for balancing efficiency with accuracy. This enabled the transformation of extracted pain and gain points into actionable and readable insights.

## Implementation and results

This section explains the experimental setup and evaluation metrics used to assess the performance of the proposed system. It concludes with a discussion of the results, including sentiment analysis, topic modeling, and summarization-based gain/pain point generation.

### Experimental setup

The proposed model’s experiments were conducted on Google Colab Pro using cloud-based hardware equipped with an NVIDIA L4 GPU (16 GB VRAM, 25 GB system RAM). Early fine-tuning and evaluation were performed on the L4 GPU, while the final BERT-Base model was trained on a v2-8 TPU for approximately 10 h over 15 epochs with a batch size of 16. RoBERTa-Base and BERT-Small were trained on Dataset2 for varying numbers of epochs. All implementations were carried out in Python 3.9.5 using PyTorch v1.8.0a0, with the AdamW optimizer and experiment-specific learning rates (e.g., ${3 \times 10^{-5}}$ for the final BERT-Base). Reporting these hardware settings, runtime configurations, and training parameters ensures the reproducibility of the results across all experimental setups.

#### Preprocessing model

A preprocessing model (e.g., en_uncased_preprocess) that is particular to BERT was used to process cleaned text by tokenizing it into the subwords & assigning numeric token IDs and appending [CLS] and [SEP] tokens as required for BERT input.

#### Hyperparameter tuning

The hyperparameter values for each model were selected through empirical testing and aligned with standard configurations used in previous transformer-based sentiment analysis studies. Lower learning rates were chosen to ensure stable fine-tuning, while batch sizes were adjusted according to hardware capacity to maintain efficient training. The number of epochs was determined experimentally to minimize overfitting, with early stopping applied once validation performance stabilized. The finalized settings for BERT-Small, BERT-Base, and RoBERTa-Base are presented in Table  4.

**Table 4:** Hyperparameter settings for each model.

Parameter	BERT-small	BERT-base	RoBERTa-base
Epochs	15 (Dataset 1) / 8 (Dataset 3)	15	5
Learning rate	AdamW default (not specified)	3$\times$10$^{-5}$	1$\times$10$^{-6}$
Optimizer	AdamW	AdamW	AdamW (weight decay 1$\times$10$^{-2}$)
Batch split / ratio	70% Train / 15% Validation / 15% Test	80% Train / 20% Validation	70% Train / 15% Validation / 15% Test
Hardware used	Google Colab L4 GPU	Google Colab Pro v2-8 TPU	PyTorch DataLoader on Colab
Model Description	4 layers, 512 hidden units, 8 attention heads; overfitting after epoch 8	12 layers, 768 hidden units, 12 attention heads; early stopping after 5 epochs without improvement	Used CrossEntropyLoss; validated per epoch on accuracy and loss

#### Fine-tuning BERT for sentiment analysis

The initial experiment used Dataset1 (10,000 reviews) and the smaller BERT model described in Section *(Sentiment Analysis Methodology)*. The model was trained for 15 epochs using the AdamW optimizer and binary cross-entropy loss with from_logits=False. Binary accuracy was the evaluation metric. Training loss and accuracy were 0.5730 and 66.78% at the first epoch, respectively, whereas accuracy and validation loss were 75.38% and 0.4856. By Epoch 15, training loss dropped to 0.0940 with 96.63% accuracy. Validation loss increased to 0.9527 with 79.45% accuracy, indicating overfitting despite improved performance. Two BERT variants were used: BERT-Small (4 layers, 512 hidden units, 8 attention heads) and BERT-Base (12 layers, 768 hidden units, 12 attention heads)

The model was retrained on Dataset1 without stemming and lemmatization to preserve the original context. It was trained for 11 epochs using AdamW. Training loss and accuracy were 0.5383 and 71.07% at the first epoch, respectively, whereas accuracy and validation loss were 79.12% and 0.4518. By Epoch 11, the training loss reduced to 0.1066 with the accuracy of 96.23%, while the validation loss rose to 0.9342 & the accuracy increased to 80.75%. Due to limited accuracy gains, a larger dataset (Dataset2, combining Threads and Instagram reviews) was used. Fine-tuning was performed over 15 epochs using an L4 GPU in Google Colab. Training time increased due to the expanded dataset. Dataset3 was trained on the same BERT-Base model. At Epoch 1, training loss was 0.4995 with 75.07% accuracy; validation loss and accuracy were 0.4159 and 81.54%. An EarlyStopping callback monitored validation loss, halting training after 5 epochs without improvement. Training stopped at Epoch 8 with a training loss of 0.1741 and accuracy 93.62%, and a validation loss of 0.5959 with 81.61% accuracy. Since Dataset2 showed better performance, all remaining experiments were conducted using it (20,000 reviews).

The BERT-Base model (12 layers, 768 hidden units, 12 attention heads)^76^ was used for its ability to capture complex semantic relationships, although with a higher computational cost. Compared to BERT-Small (4 layers, 512 hidden units, 8 attention heads), BERT-Base offered enhanced accuracy. A pre-trained version from TensorFlow Hub was used with a custom classifier (a dense layer with 256 units and ReLU, followed by a sigmoid output). Final training used Google Colab Pro with a v2-8 TPU for 15 epochs, taking about 10 h. The learning rate ${3x10^{-5}}$ remained. To preprocess text embeddings with the Transformer encoders, the preprocessing model “en_uncased_preprocess”^77^ was used. This model processes one or more batches of plain UTF-8 encoded text segments and transforms them into inputs compatible with the Transformer encoder, offering an efficient method for preparing data for the encoder.

#### RoBERTa implementation

RoBERTa-Base and BERT-Small were fine-tuned for 5 epochs on Dataset2. Each model used its respective tokenizer. The data was split into 70% training, 15% validation, and 15% testing using train_test_split with random_state=42. The pre-trained roberta-base tokenizer converted text into numerical input. Each review was tokenized and padded/truncated to a max length of 128 tokens, then passed to PyTorch DataLoader for efficient batching. RoBERTa was fine-tuned using the AdamW optimizer (learning rate $1\times 10^{-6}$, weight decay $1\times 10^{-2}$). Loss was computed using CrossEntropyLoss. Model performance was evaluated after each epoch based on training and validation loss and accuracy. The model was tested on the reserved 15% test set. Evaluation included accuracy, precision, recall, F1-score, and a confusion matrix to analyze classification effectiveness. To demonstrate practical application, real review samples were fed into fine fine-tuned RoBERTa model. The model correctly classified these comments, confirming its capability in sentiment analysis.

#### Topic modeling with LDA

LDA was applied to discover hidden topics. Preprocessing included changing text to lowercase & eliminating stop words and non-alphabetic letters. A document-term matrix (DTM) was generated using CountVectorizer with max_df=0.95 and min_df=2. LDA outputs included representative words per topic, visualized through word clouds. Dominant topics across documents were also identified. Adjusting the value of n_components allowed the level of detail in the topics to be adjusted, making the analysis either more general or more detailed depending on the dataset. The parameter was tested with three settings: n_components=10 to produce ten topics, n_components=20 to produce twenty topics, and n_components=30 to produce thirty topics.

#### Text summarization for pain points and gain points

Summarization and sentence generation were conducted using Paperguide.ai, Scribbr, and ChatGPT, based on the 30 LDA-identified topics. Both extractive and abstractive summarization methods were applied. ChatGPT provided the best results, generating coherent, relevant summaries from large datasets. Results are shown below for a selected set of five negative topics. Built-in tools were used to summarize the text and generate the final output in the form of sentences or phrases. The usage of both AI and non-AI tools demonstrated how numerous tools condense the topics for a final output. Results from ChatGPT were the best.

*Original negative topics*

1. people, app, use, doesnt, dont, instagram, update, post, fix, account;
2. people, data, app, follow, useless, downloaded, feed, download, working, dont;
3. instagram, app, account, new, dont, reply, reason, work, insta, ive;
4. follow, data, people, app, useless, dont, feed, download, working, downloaded;
5. better, twitter, app, version, facebook, think, frustrating, update, tiktok, right;
6. tried, im, fix, story, reel, instagram, unable, app, video, update.

*Paperguide.ai results*

1. People reported that Instagram posting issues were not fixed by account updates.
2. I can’t get into my Instagram account. Instagram hasn’t responded to me about this issue.
3. Facebook users are frustrated by the improved Twitter app.
4. The inability to download worthless data from an inoperable app prevents users from following others.
5. Unable to resolve video reel stories and Instagram app updates.

*Scribbr results*

1. Users of the Instagram app don’t resolve bugs. They update their accounts, upload content, and address issues.
2. I’ve been using Instagram for a long, but I don’t know why I can’t respond to my new account when I don’t have one.
3. Facebook users may find the Twitter app’s update more aggravating than the prior version, according to the user.
4. Although the software functions properly, users find it useless to download and follow.
5. The Instagram app is giving users trouble, making it impossible to upgrade, and malfunctioning. They’re working to resolve the problem and enhance their profile.

*ChatGPT results*

1. Instagram’s app is malfunctioning; accounts and posts need to be fixed.
2. The Instagram account is not functioning, and no reply from support.
3. Updates on Twitter and TikTok seem superior to those on Instagram.
4. App feed is useless; downloaded but not working.
5. Unable to resolve problems with Instagram stories and reels after updating.

ChatGPT’s built-in algorithm is used to generate its results. Using transformer-based models akin to GPT, the task employs abstractive summarization, which rewords text while maintaining meaning. In order to generate meaningful and succinct sentences, it integrates semantic analysis with context understanding. ChatGPT was given the input to turn the topics into sentences that are shown in as https://chatgpt.com/share/6787a244-0828-800e-bfb5-a48fb9c03c9f.

### Evaluation metrics

To assess the performance of the sentiment analysis models, several evaluation metrics are used, including Accuracy, Precision, Recall, a customized F1 Score, Specificity, and False Negative Ratio (FNR). These metrics together allow a comprehensive evaluation of model performance in both binary and multi-class sentiment analysis tasks.

#### Accuracy

Accuracy measures the proportion of total correct predictions out of all predictions made. While it offers a basic evaluation, it can be misleading for imbalanced datasets where one class may dominate. It is computed as:

1 $$\begin{aligned} Accuracy = \frac{TP+TN}{TP+TN+FP+FN}. \end{aligned}$$

#### F1-score

The F1 Score provides a harmonic mean between Precision and Recall, making it useful when there is an uneven class distribution or where both false positives and false negatives carry a cost. The customized F1 Score adopted in this research is defined as:

2 $$\begin{aligned} F1 = \frac{2*Precision*Recall}{Precision+Recall} = \frac{2*TP}{2*TP+FP+FN}. \end{aligned}$$

#### Precision

Precision is the fraction of correctly predicted positive observations out of all predicted positives. It is essential in cases where false positives are costly. Precision is calculated as:

3 $$\begin{aligned} Precision = \frac{TP}{TP+FP}. \end{aligned}$$

#### Recall

Recall, also known as Sensitivity or True Positive Rate, is the fraction of actual positives that are correctly predicted by the model. It becomes critical when the cost of missing positive instances (false negatives) is high:

4 $$\begin{aligned} Recall = \frac{TP}{TP+FN}. \end{aligned}$$

These combined metrics offer a well-rounded assessment of the model’s predictive ability, especially in the context of sentiment classification, where class imbalance, contextual nuance, and trade-offs between false positives and false negatives are common.

## Experimental results

### Sentiment analysis results

#### BERT-small

**Fig. 7:**
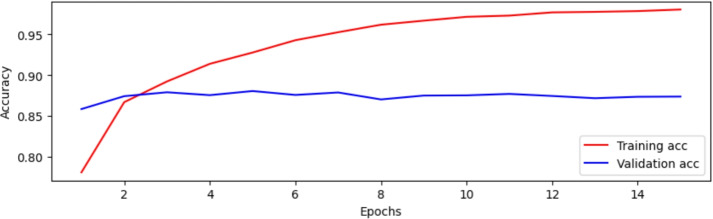
Comparison of the BERT-Small model’s accuracy during the training and validation stages over 15 epochs.

The BERT-Small model, trained on Dataset 2, showed promising results. In the first epoch, a binary accuracy of 78.14% and a training loss of 0.4485 were achieved. The accuracy of the validation was 85.87%, and the validation loss was 0.3280. As the training progressed, the model improved significantly in terms of training accuracy, reaching a training loss of 0.0595 and binary accuracy of 98.04% at Epoch 15. However, the validation loss increased to 0.7483, and the validation accuracy reached only 87.40%, indicating signs of overfitting. This behavior is illustrated in Fig. 7, which presents the trend of training and validation accuracy over epochs. The performance plateaued after the 8th epoch, suggesting limited gains beyond that point. The overfitting observed in BERT-Small resulted from the limited training corpus relative to model parameters. It was mitigated through early stopping, a 0.3 dropout layer, and training on augmented datasets.

#### BERT-base

**Fig. 8:**
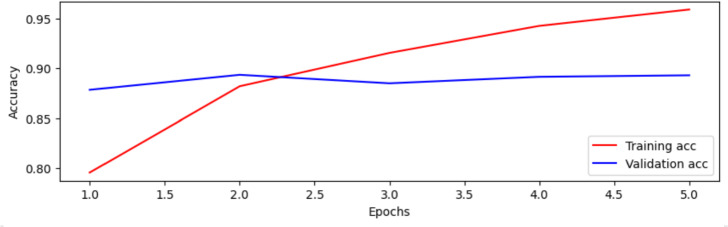
Comparison of the BERT-Base model’s accuracy during the training and validation stages over 5 epochs.

The BERT-Base model was trained for 5 epochs on Dataset 2. A binary accuracy of 79.59% and training loss of 0.4300 were achieved in the first epoch. The accuracy of the validation was 87.85%, and the validation loss was 0.3000. After 5 epochs, training loss decreased to 0.1289, and the binary accuracy improved to 95.89%. The validation accuracy also improved to 89.30%. BERT-Base performed better than BERT-Small due to its increased depth, number of attention heads, and hidden layers. Figure 8 shows the progression in accuracy across training and validation phases.

#### Standard BERT-base (final model)

**Fig. 9:**
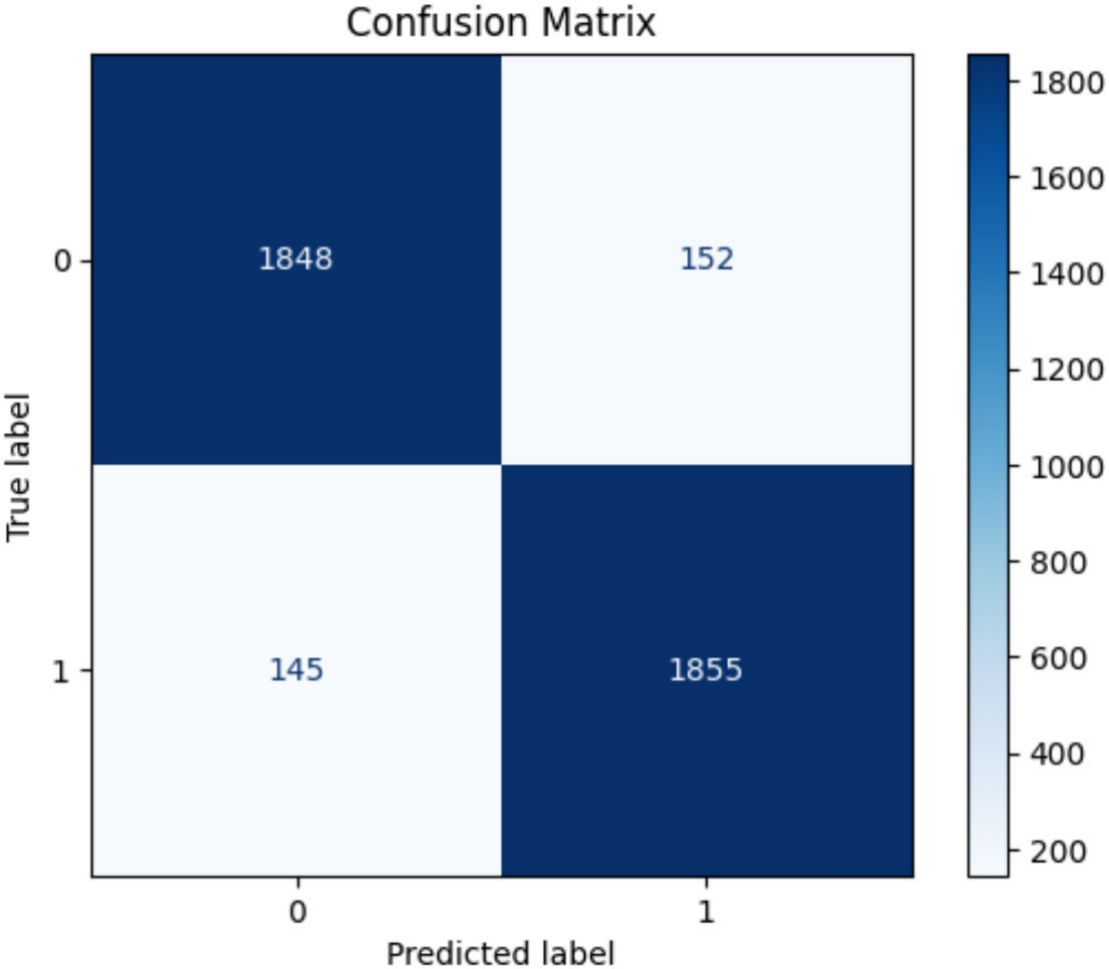
Confusion matrix illustrating the final BERT-Base model’s classification performance, showing both correct and incorrect predictions for each sentiment category.

**Fig. 10:**
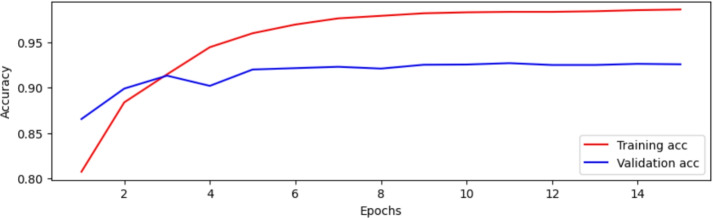
Learning curve of final BERT-Base model’s accuracy during the training and validation stages over 15 Epochs.

The final sentiment analysis experiment utilized the Standard BERT-Base model. The model’s performance at Epoch 1 was as follows: training loss of 0.4131, precision of 83.24%, binary accuracy of 80.75%, F1 score of 80.00% and recall of 77.00%. The validation set showed an accuracy of 86.55%, recall of 91.80%, precision of 83.08%, F1 score of 87.22%, and a loss of 0.3536. By Epoch 15, training accuracy reached 98.61%, with a loss of 0.0385, precision of 97.82%, F1 score of 98.62% and recall of 99.42%. The validation metrics also peaked at 92.58% accuracy, 92.43% precision, 92.75% recall, and 92.59% F1 score. Figure 9 illustrates the confusion matrix, and the learning curve is shown in Fig. 10.

#### RoBERTa-base

RoBERTa-Base showed strong performance in sentiment classification. During the initial epoch, it achieved a training accuracy of 74.91% and a validation accuracy of 89.30%. At Epoch 5, the training accuracy increased to 91.86%, while the validation accuracy reached 90.07%. Although it outperformed BERT-Small in validation metrics, its overall results were slightly below those of the Standard BERT-Base model.

#### Model comparison summary

Table  5 compares the performance of all fine-tuned models. The Standard BERT-Base model achieved the highest accuracy and F1 score. RoBERTa-Base followed closely, while earlier BERT-Small experiments revealed overfitting, which was mitigated by adjusting the preprocessing techniques.

**Table 5:** Evaluation of fine-tuned model performance.

Model	Dataset	Preprocessing	Epochs	Accuracy (%)	F1-score (%)	Remarks
BERT-Small (v1)	Dataset 1	With stemming & lemmatization	15	79.45	$\sim$ 79	Overfitting observed
BERT-Small (v2)	Dataset 1	Without stemming & lemmatization	11	80.75	$\sim$ 80	Improved generalization
BERT-Small (v2)	Dataset 3	Without stemming & lemmatization	8	81.61	$\sim$ 81	Larger dataset improved results
Standard BERT-Base	Dataset 2	en_uncased_preprocess	15	92.58	92.59	Best performance, selected final model
RoBERTa-Base	Dataset 2	roberta-base tokenizer (128 tokens)	5	$\sim$ 90	90.60	Competitive but slightly below standard BERT-Base

### Topic modeling results

The two categories of topic modeling results from LDA are positive and negative topics, which are then utilized for gain and pain points.

#### Negative topics

**Fig. 11:**
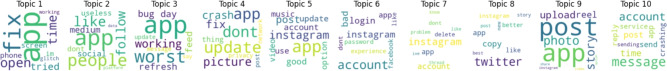
A word cloud representation for ten negative sentiment topics that were found via topic modeling analysis.

**Fig. 12:**
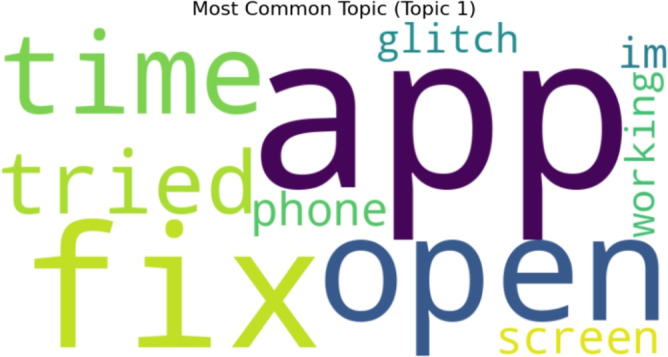
Word cloud representation of the most frequent topic among the ten negative topics generated using topic modeling.

Figure 11 displays a word cloud of keywords that were retrieved by topic modeling for ten negative sentiment topics. Topic number 1 is the most popular topic, as shown in Fig. 12.

**Fig. 13:**
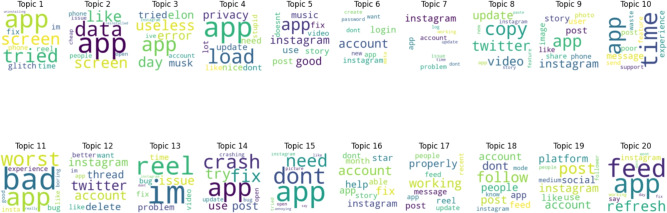
A word cloud representation for twenty negative sentiment topics that were found via topic modeling analysis.

**Fig. 14:**
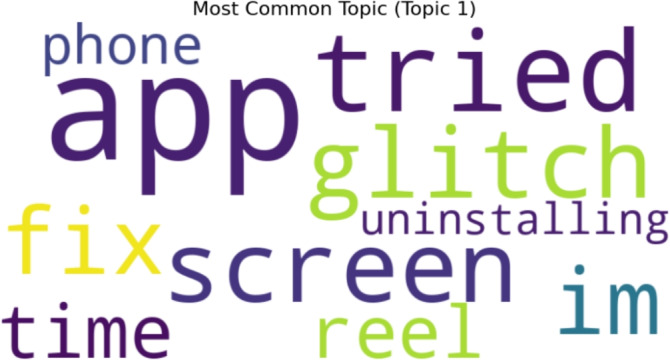
A word cloud representing the most frequent topic among the twenty negative topics generated using topic modeling.

**Fig. 15:**
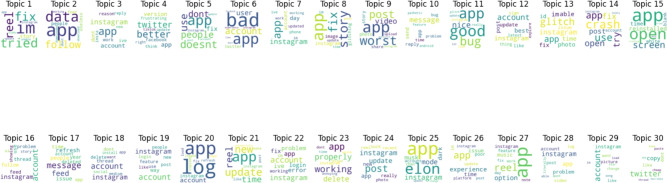
A word cloud representation for thirty negative sentiment topics that were found via topic modeling analysis.

**Fig. 16:**
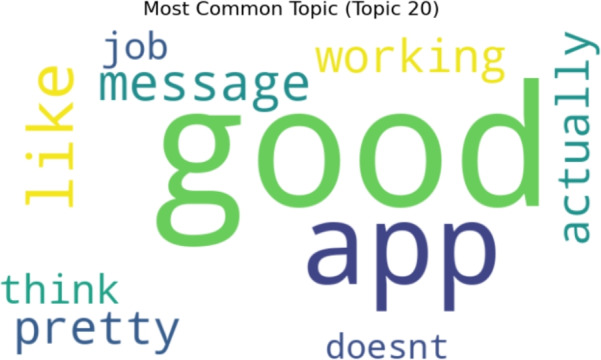
A word cloud representing the most frequent topic among the thirty negative topics generated using topic modeling.

Figure 13 displays outcomes of twenty negative topics. As seen in Fig. 14, Topic 1 is the most frequently discussed of them. According to Fig. 15, which displays the findings of thirty negative sentiment topics, Topic 20 is the most prevalent, as seen in Fig. 16.

#### Positive topics

**Fig. 17:**
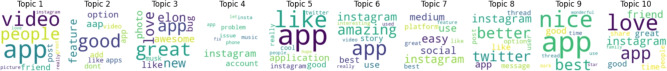
A word cloud representation for ten positive sentiment topics that were found via topic modeling analysis.

**Fig. 18:**
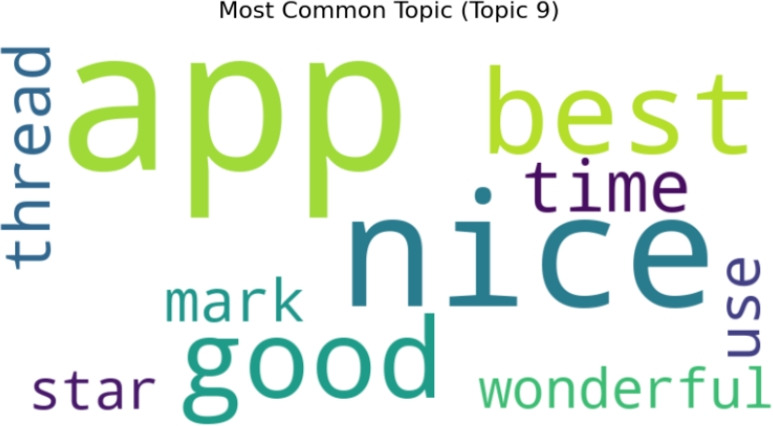
A word cloud representing the most frequent topic among the ten positive topics generated using topic modeling.

Word cloud visualization for ten topics generated by LDA topic modeling, showing the most prevalent and typical terms for each topic. In Fig. 17, the findings for the ten positive topics are shown. Topic 9 is the most common, as illustrated in the Fig. 18.

**Fig. 19:**
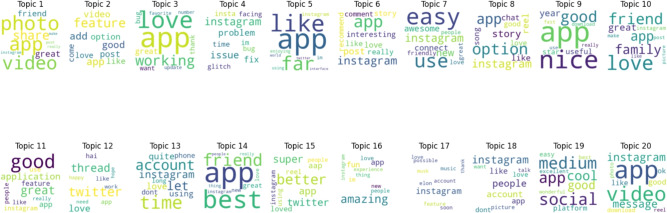
A word cloud representation for twenty positive sentiment topics that were found via topic modeling analysis.

**Fig. 20:**
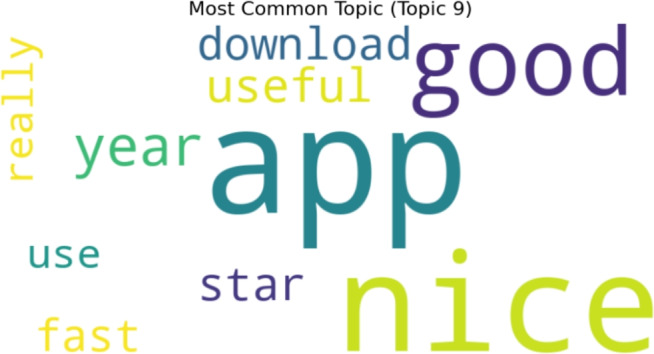
A word cloud representing the most frequent topic among the twenty positive topics generated using topic modeling.

Word cloud visualization for twenty topics generated by LDA topic modeling. The most prevalent and typical terms taken from the dataset are shown for each topic. Figure 19 presents the findings of twenty topics, with Topic 9 being the most prevalent, as seen in Fig. 20.

**Fig. 21:**
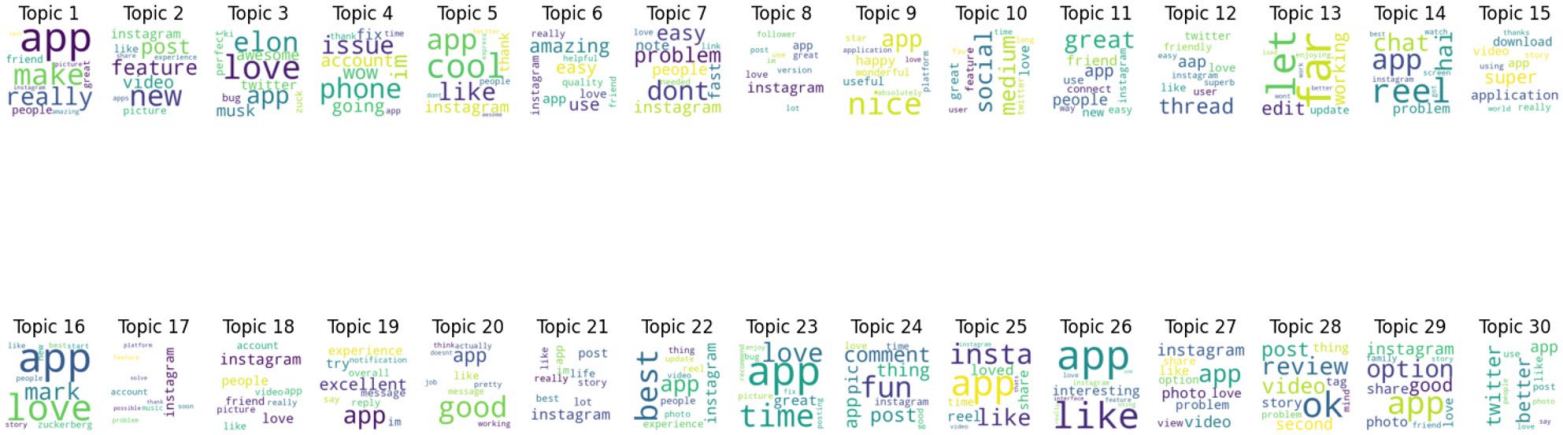
A word cloud representation for thirty positive sentiment topics that were found via topic modeling analysis.

**Fig. 22:**
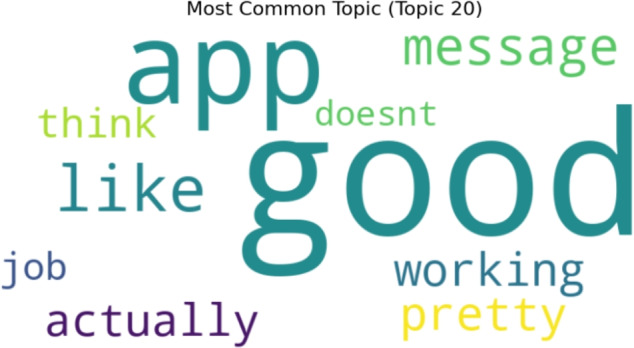
A word cloud representing the most frequent topic among the thirty positive topics generated using topic modeling.

Figure 21 presents the results of 30 positive sentiment topics, with Topic 20 being the most common, as seen in Fig. 22.

### Gain points final output

The findings of this study, presented in tabular and visual formats, highlight gain points derived from positive user feedback. These gain points were extracted using topics generated through LDA-based topic modeling and include aspects such as usability, ease of access to resources, clear processes or interfaces, timely support, and satisfaction when expectations are met. These themes represent key areas where users reported positive experiences during their interaction with the apps. The identified gain points were further processed through ChatGPT to generate concise, readable sentence-level summaries of the topics for improved interpretability. These summarized gain points are illustrated in Table  6.

**Table 6:** Positive topics and gain points output.

Topic no.	Input (raw topic data)	Output (summarized text i.e., gain points)
1	app, make, really, people, friend, great, picture, amazing, instagram, text	Instagram is great for making friends and sharing pictures
2	new, feature, post, video, instagram, picture, like, experience, apps, share	New Instagram features improve video and picture sharing
3	love, elon, app, musk, awesome, twitter, perfect, bug, zug, ki	Elon Musk’s app is awesome but needs bug fixes
4	phone, issue, im, wow, going, account, fix, thank, app, time	Fix phone issues and account problems in less time
5	cool, app, like, instagram, thank, people, twitter, express, dont, awsome	Cool app to express and connect with people on Instagram
6	amazing, use, easy, app, love, instagram, quality, really, friend, helpful	Instagram is amazing, easy to use, and helpful for friends
7	dont, problem, easy, instagram, fast, people, note, needed, love, link	Instagram is fast and easy to use, loved by many
8	instagram, app, love, great, follower, version, post, lot, use, im	Great app for posts and connecting with followers
9	nice, app, happy, useful, wonderful, star, platform, absolutely, application, love	Wonderful platform for sharing and staying connected
10	social, medium, love, great, feature, twitter, user, long, time, fav	Twitter is a favorite social media platform for users
11	great, app, people, friend, new, use, connect, instagram, easy, way	Instagram connects people and friends in an easy way
12	thread, aap, love, twitter, like, user, friendly, instagram, superb, easy	Threads app is user-friendly and superb for social media
13	far, let, edit, working, update, enjoying, wont, load, work, better	Update needed for better app functionality and editing
14	reel, app, chat, hai, problem, instagram, screen, watch, got, best	Instagram reels are great, but screen issues need fixing
15	super, application, download, video, app, really, story, using, thanks, world	Download the app for videos and sharing stories worldwide
16	app, love, mark, zuckerberg, new, people, story, best, like, start	Mark Zuckerberg’s new app is great for stories and people
17	instagram, account, music, soon, problem, feature, platform, solve, possible, thank	Fix Instagram music problems soon for a better platform
18	instagram, love, people, friend, account, app, like, video, picture, really	Instagram is loved for sharing videos and pictures with friends
19	app, excellent, try, experience, im, message, overall, reply, say, notification	Excellent app for messaging and overall experience
20	good, app, like, message, pretty, working, actually, think, job, doesnt	Good app for messaging, but some issues need fixing
21	instagram, post, app, life, im, lot, best, story, like, really	Instagram posts and stories add fun to life.
22	best, app, instagram, experience, people, reel, thing, photo, update, video	Instagram reels and videos offer the best experience
23	app, time, love, great, picture, bug, posting, recommend, fix, enjoy	Fix bugs to enjoy posting pictures on the app
24	fun, comment, post, thing, app, pic, instagram, time, love, good	Instagram is fun for posting pics and commenting
25	app, insta, like, loved, reel, share, time, instagram, video, thats	Instagram reels and videos are great for sharing.
26	app, like, interesting, instagram, love, feature, using, interface, really, use	Love Instagram’s interesting features and user-friendly interface
27	app, instagram, video, photo, problem, like, love, option, share, view	Instagram is great for sharing videos and photos
28	ok, review, video, post, second, story, thing, tag, problem, mind	Review Instagram’s tagging issues in video posts
29	app, option, good, instagram, share, photo, love, family, friend, story	Instagram options are good for sharing family stories
30	twitter, better, app, like, use, post, love, photo, people, say	Twitter is better for posting photos and connecting people

### Pain points final output

Pain points were identified using the same LDA-based topic modeling process, focusing on negatively classified reviews. These topics highlight issues such as usability challenges, limited access to resources, confusing interfaces or processes, lack of timely support, and frustration when expectations were not met. To enhance interpretability, ChatGPT was used to convert these topics into concise sentence or phrase summaries. The resulting pain points, presented in Table  7, have direct implications for UX design: they reveal usability issues that require redesign or refinement, while gain points as shown in Table  6 emphasize features that enhance user satisfaction and should be preserved or strengthened in future updates.

**Table 7:** Negative topics and pain points output.

Topic no.	Input (raw topic data)	Output (summarized text i.e., pain points)
2	follow, app, people, data, useless, dont, feed, download, working, downloaded	Feed not working, app should be avoided
3	account, app, dont, instagram, work, reason, ive, insta, new, reply	Instagram account not working, no support for issues
4	app, better, version, twitter, facebook, frustrating, think, update, right, tiktok	Instagram update frustrating, Twitter is better than Facebook and TikTok
5	use, app, people, doesnt, dont, post, fix, account, update, instagram	App issues not fixed, while posting on Instagram
6	bad, app, account, user, twitter, experience, instagram, dont, like, facebook	Bad user experience, prefers Twitter over Instagram and Facebook
7	app, instagram, day, account, phone, ive, updated, im, work, working	Instagram app not working after update, phone issues
8	app, story, fix, instagram, image, glitching, im, update, post, time	Instagram story glitches and image issues after update
9	app, worst, post, video, photo, story, share, upload, instagram, application	Worst app for uploading posts, photos, and videos on Instagram
10	message, send, app, reply, bug, time, problem, android, feature, pathetic	Bug in sending messages and replying on Android app
11	good, app, bug, nice, instagram, really, like, glitch, update, lot	Good app with bugs and glitches on Instagram, needs update
12	account, instagram, best, post, update, like, sign, thing, latest, explore	Instagram account update issues, poor post functionality
13	glitch, app, instagram, im, able, time, photo, fix, id, issue	Instagram app glitch, photo upload issue
14	crash, use, app, try, open, fix, post, account, instagram, lately	Instagram app crashing, post functionality not working
15	app, open, screen, reinstalled, time, white, blank, crashing, tried, work	Instagram app shows white screen, crashes persist after reinstall
16	account, instagram, use, thread, follow, feed, problem, ui, showing, story	Instagram feed and threads not working, UI issues
17	message, feed, refresh, issue, people, year, time, deleted, account, app	Feed not refreshing, account deletion issue
18	account, instagram, thread, delete, dont, social, want, medium, install, app	Instagram thread deletion issue, prefers other social apps
19	account, instagram, way, feature, post, new, use, people, login, like	Instagram login and feature issues
20	log, app, account, instagram, fix, copied, load, refresh, feed, logged	Instagram feed not refreshing, account login issues
21	app, update, new, reel, time, instagram, like, make, post, automatically	Instagram reel and post issues after update
22	account, instagram, error, app, ive, problem, fix, login, working, im	Instagram login error, app not working properly
23	working, app, properly, delete, instagram, dont, time, annoying, waste, thread	Instagram app not working, thread issues, waste of time
24	post, instagram, update, app, new, really, suck, photo, reel, recent	Instagram update causing post, reel, and photo problems
25	app, elon, instagram, mode, musk, dark, account, chat, update, change	Instagram dark mode causing issues, update problems
26	app, experience, time, issue, poor, im, update, platform, instagram, post	Poor experience on Instagram, update and post issues
27	app, reel, option, music, instagram, feature, note, work, fix, day	Instagram reel music feature not working, needs fixing
28	instagram, app, problem, account, post, video, log, wont, able, literally	Instagram login and post issues, unable to upload
29	account, instagram, picture, like, want, change, im, load, star, song	Instagram profile picture not loading, needs change
30	twitter, copy, dont, like, paste, cheap, thread, know, horrible, app	Issues with copying and pasting in Threads app, prefers Twitter

### Discussion on empathy map quadrants and UX insights

The empathy mapping process in this study followed the four standard quadrants: Thinks, Feels, Says, and Does. While the analysis mainly emphasized the Pain and Gain areas, these are closely related to the main empathy map dimensions. The Thinks and Feels quadrants reflect what users believe or experience internally, often revealed through negative feedback such as issues with the feed, login problems, or poor performance. The Says and Does quadrants capture outward expressions and actions, which were mostly reflected in positive experiences such as sharing posts, exploring new features, or engaging with friends. Relating the extracted pain and gain points to these four quadrants helps illustrate how user emotions connect with behavior and interaction patterns, providing practical guidance to improve the overall app experience.

### Comparison with baseline state-of-the-art models

In this section, the performance of the proposed BERT-Base model was evaluated in comparison with several traditional state-of-the-art models, including SVM, Naïve Bayes (NB), and LSTM, to establish a reliable performance benchmark for sentiment classification on app reviews. The results are summarized in Table  8, which presents the F1-score and accuracy values for each model. The proposed BERT-Base (fine-tuned) model achieved the highest results, with an F1-score of 92.59% and an accuracy of 92.58%, showing a clear improvement over all baseline approaches.

The Naïve Bayes classifier obtained relatively lower performance (F1 = 77.1%, Accuracy = 78.2%) because it relies mainly on word-frequency features and does not effectively handle the informal or noisy nature of user reviews. The SVM model performed moderately better than Naïve Bayes but still fell short of the transformer models, as it cannot understand deeper contextual relationships within the text. The LSTM model (F1 = 89.0%, Accuracy = 90.0%) produced competitive results due to its sequential processing of text, yet it was surpassed by BERT-Base, which benefits from pre-trained contextual embeddings and bidirectional language understanding. Table  8 indicates that the fine-tuned BERT-Base model provides the most accurate and context-aware sentiment classification results, confirming its advantage over conventional machine learning and sequential models for analyzing app review data.

**Table 8:** Performance evaluation comparison of the proposed model with other state-of-the-art models.

Classifier	F1-score (%)	Accuracy (%)
Support vector machine (SVM)	83.0	84.1
Naïve Bayes (NB)	77.1	78.2
Long short-term memory (LSTM)	89.0	90.0
**BERT-small**	**81.0**	**81.61**
**Standard BERT-base**	**92.59**	**92.58**
**RoBERTa-base**	**90.60**	**90.0**

### Discussion

The experimental results provide comprehensive insights into the performance of transformer-based models for sentiment analysis of app reviews. Among the tested models, BERT-Base achieved the highest validation accuracy (92.58%) and F1 score (92.59%), demonstrating strong generalization due to its deep architecture and effective preprocessing pipeline. RoBERTa-Base also performed competitively, though it required more data and training epochs to match BERT-Base consistently, reflecting its reliance on optimized pretraining. Experiments with BERT-Small revealed vulnerabilities such as overfitting, particularly when preprocessing techniques like stemming and lemmatization were applied. Refinements to the preprocessing pipeline and expansion of training datasets improved validation accuracy to 81.61%, but the model remained less effective than its deeper counterparts. These findings underscore the combined importance of architectural complexity and appropriate data treatment in sentiment classification tasks. The customized BERT layers provide interpretability by revealing language patterns linked to user emotions and usability concerns. These insights enable UX designers to recognize common user issues, set priorities for design improvements, and ensure that interface changes align with user expectations. For instance, strong attention weights on words like “navigation” or “loading time” point to specific aspects of the interface that may be causing frustration.

Although the deeper transformer models achieved higher accuracy, they also required substantially more computational resources. The greater number of attention layers in BERT-Base increased training time and memory usage, making it less efficient to run compared with smaller models such as BERT-Small. RoBERTa-Base also demanded additional computation because of its extended pre-training and fine-tuning stages. These results point to a clear trade-off between accuracy and efficiency: models with more attention layers provide stronger contextual understanding but are costlier to train and deploy. Choosing the right configuration, therefore, depends on available hardware resources and the scale at which the system is intended to operate.

Topic modeling played a vital role in enhancing the interpretability of sentiment analysis results. The LDA word clouds provided a clear visualization of the dominant themes in sentiments. For negative reviews, key issues included app crashes, login difficulties, and inadequate customer support. Positive topics focused on usability, performance, and interface design. Notably, some gain points included terms typically associated with negative sentiment. This indicates that even positively rated reviews sometimes highlight lingering issues. These mixed sentiments emphasize the need for nuanced analysis: positive ratings can coexist with complaints, and seemingly negative words may appear in constructive contexts. Summarization using ChatGPT helped convert abstract topic clusters into human-readable insights, making gain and pain points more accessible for empathy mapping and user experience design. For instance, frequent complaints about “story glitches” indicate a need to optimize video-buffering algorithms, whereas positive comments on “ease of content sharing” justify maintaining and extending existing sharing workflows. Increasing the number of topics (from 10 to 30) allowed for more granular analysis, though it occasionally introduced redundancy, highlighting the balance needed in topic resolution.

Overall, the results validate the efficacy of fine-tuned BERT-based models, particularly BERT-Base, in sentiment classification. When paired with unsupervised topic modeling and summarization, this approach offers a robust framework for deriving actionable UX insights from user-generated content. The proposed framework can be extended to other domains such as e-commerce, education, and healthcare apps to evaluate its adaptability across distinct user populations.

A limitation of this study is that it relies only on app reviews, which may not fully capture the deeper thoughts or emotions that could be revealed through interviews or surveys. Future work will combine these methods to develop a more comprehensive understanding of empathy mapping. In future work, this approach can be extended to multilingual and cross-platform datasets, such as reviews from different app ecosystems, to evaluate the generalizability of the pain/gain extraction and empathy mapping framework across various user groups.

## Conclusion

This study demonstrates that BERT-based sentiment analysis combined with LDA topic modeling can effectively generate empathy maps from large-scale social media app reviews. The approach achieved strong performance, showing that enhanced attention layers improve accuracy on larger datasets, though with higher computational cost. Using the combined Instagram and Threads datasets provided richer and more diverse insights, confirming the value of broader data sources.

The work contributes a practical data-driven framework to identify user pain and gain points, supporting UX design and persona development. However, the model currently captures only surface-level sentiment and requires further refinement to reflect deeper emotional and cognitive aspects of user experience. Future work will focus on expanding the framework to include additional empathy map quadrants, such as Thinks and Feels, and on integrating data from interviews, app store reviews, and other social media sources to improve emotional depth and interpretive accuracy. This expansion will help capture richer UX perspectives and better meet evolving user expectations.

## Data Availability

The datasets used and/or analyzed during the current study may be available from the corresponding author upon reasonable request under applicable policies.
